# Canonical Wnt signaling regulates Mbd3 protein stability during neurogenesis

**DOI:** 10.1038/s12276-025-01510-4

**Published:** 2025-08-01

**Authors:** Nhu Thi Quynh Mai, Soyoung Jeon, Byoung-San Moon

**Affiliations:** 1 Department of Medical Biotechnology, Yeungnam University, Gyeongsan, Republic of Korea; 2 Center for Genetic Epidemiology, Department of Population and Public Health Sciences, Keck School of Medicine, University of Southern California, Los Angeles, CA USA; 3 Cancer Biology and Genomics Graduate Program, Program in Biological and Biomedical Sciences, Keck School of Medicine, University of Southern California, Los Angeles, CA USA

**Keywords:** Cell signalling, Neurogenesis, Neural stem cells

## Abstract

Acquisition of neural progenitor cell (NPC) homeostasis through balancing self-renewal and differentiation is essential for brain development and function. Among the mechanisms controlling these processes, canonical Wnt signaling and the Mbd3–NuRD complex, with prominent suppressive effects on neurogenesis, have been described as crucial parts of the core regulatory circuit. Here we explored Mbd3 as a downstream element of the canonical Wnt signalosomes. Specifically, dynamic modulation of Wnt signaling through activator (Wnt3a) and inhibitor (DKK1) resulted in parallel alterations in β-catenin and Mbd3 expression patterns. Also, overexpression and depletion of GSK3β respectively promoted and attenuated Mbd3 ubiquitination, highlighting that the canonical Wnt cascade promotes Mbd3 stability. Downstream of the Wnt–β-catenin pathway, Mbd3 represses transcription of neurogenesis-associated genes by triggering NuRD complex assembly, thereby promoting NPC stemness. This new Wnt–Mbd3 axis extends the current understanding of the canonical Wnt network in directing neuronal cell-fate determination in NPCs, suggesting this pathway as a potential target for driving neural stem cell reprogramming and neuronal lineage commitment.

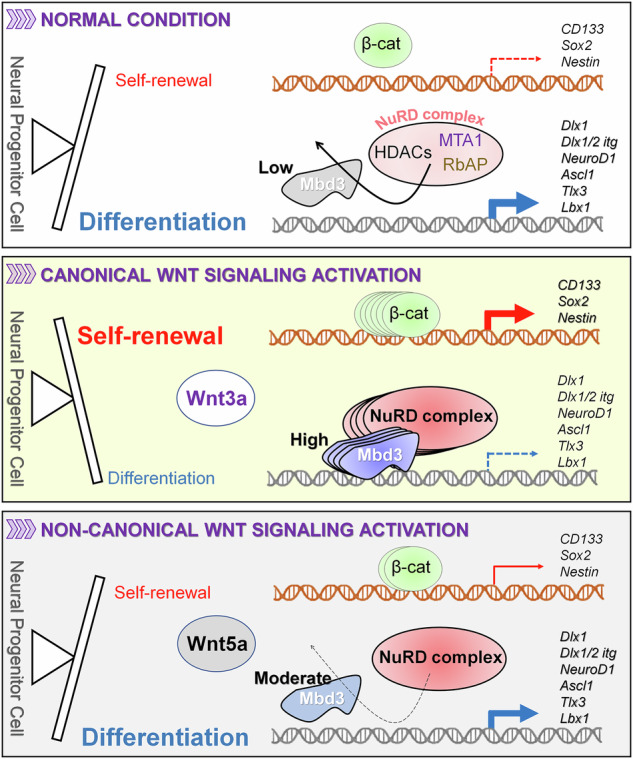

## Wnt–Mbd3 axis regulates neural progenitor cell fate

Neurogenesis—the process of generating new brain cells—is essential for healthy brain function. To support this, the brain must balance the growth of neural stem cells with their differentiation into specialized brain cells. This study uncovers a pivotal role for Mbd3 protein in regulating that balance. The researchers demonstrated that activation of Wnt signaling stabilizes Mbd3, enabling it to participate in assembling a protein complex that temporarily suppresses genes involved in stem cell differentiation. This suppression helps preserve the brain’s pool of neural stem cells. By elucidating the link between Wnt signaling and Mbd3 in regulating neurogenesis, the findings provide important insights into brain development and the maintenance of neural homeostasis. This research also opens potential therapeutic avenues for treating conditions linked to stem cell dysregulation, such as neurodegenerative and neurodevelopmental disorders.

This summary was initially drafted using artificial intelligence, then revised and fact-checked by the author.

## Introduction

Neurogenesis is an intricated multistage process that is indispensable for a fully functional central nervous system^1^. During this process, neural stem cells, with self-renewal and differentiation capacity, continuously produce cells through symmetric and asymmetric division to maintain the pluripotency, and give rise to neural progenitor cells (NPCs) that generate a multitude of specialized brain cells^2^. Growing evidence has suggested that the sequential steps of neurogenesis must be tightly regulated to maintain a proper balance between proliferation and differentiation of neural stem cells, a process termed NPC homeostasis^3^. Inappropriate interference disturbing this balance could impair neurogenesis and disrupt regenerative demands, resulting in numerous neurological diseases^4,5^. These findings highlight the critical importance of maintaining NPC homeostasis during development and adult life.

So far, numerous factors, including multiple extracellular matrix components^6^, cytoplasmic factors^7^ and nuclear receptors^8^, have been indicated to coordinate the strict control of NPC proliferation and differentiation. In addition, extrinsic niche stimuli^9^ and intrinsic transcription factors^10^ have been indicated to be crucial for maintaining NPC reservoirs throughout the formation of functional neural circuits, a process largely coordinated by signaling pathways. Among the multitude of signaling pathways contributing to the maintenance of NPC homeostasis^11–13^, Wnt–β-catenin shows its profound importance by functioning as a critical regulator of NPCs homeostasis during development^14–17^. Notably, this cascade could promote both progenitor self-renewal and differentiation, determining which process to favor in a stage-specific manner. Accordingly, the canonical Wnt pathway promotes self-renewal and maintains neural progenitors during early neurogenesis^18–20^, while inducing differentiation during mid-to-late stages of neurogenesis^3,21–23^. The acquisition of these modulations, in fact, is enabled by β-catenin, a well-known inducing transcription factor that binds target genes promoters or enhancers under the upstream regulation of the canonical branch of Wnt pathway. Wnt–β-catenin has been shown to regulate the expression of various genes required for stemness or neurogenesis^16,23^. For that reason, the flexible property of the canonical Wnt pathway is characterized by its network, in which involved molecules perform their unique functions under the direction of Wnt. Studying molecules participating in this circuit would therefore deepen our understanding of the regulation and demands in the proliferation–differentiation balance during neurogenesis.

Methyl-CpG-binding domain 3 (Mbd3), a member among the five-MBD protein family, is unique in its ability to specifically recognize 5′-hydroxymethyl-cytosine (5′-hmC), an epigenetic marker that is highly enriched in stem cells and cancer^24–26^. Serving as a scaffold protein recruiting the assembly of the NuRD complex components that in turn exerts transcriptionally suppressive effects on target genes^27^, Mdb3 acts as a transcription repressor functioning during development and homeostasis^28^. Research has found high expression of Mbd3 in cortical neuroepithelial cells of the embryonic forebrain, suggesting a notable contribution of this factor during neuronal lineage commitment^29^. In support of this notion, we previously demonstrated that overexpression of Mbd3 suppresses the expression of specific preneural genes, thereby promoting the maintenance of NPC stemness during neurogenesis^30^. A factor inducing Mbd3 destabilization was also identified, revealing the regulation of this protein. For the potential contribution of Mbd3 in neurogenesis, further investigations deepening our understanding of the mechanism governing its activity are required.

Here, we investigated the correlation between Mbd3 and β-catenin expression and identified Mbd3 as a downstream component of the canonical Wnt pathway, as indicated by parallel changes in their expression patterns under conditions that activate or inhibit Wnt signaling. Through overexpression and silencing of GSK3β, a key factor inducing ubiquitin-mediated degradation, we emphasized the promoting effect of canonical Wnt signaling on stabilizing Mbd3 protein, which, in turn, enriches the occupation of the Mbd3–NuRD complex on neurogenesis-associated genes and enhances NPC stemness properties. The interaction between Mbd3 and representative subunits of the NuRD complex is enhanced under Wnt3a conditions, suggesting that canonical Wnt signaling regulates the binding affinity of Mbd3 to NuRD components, thereby facilitating its role in complex assembly. Given that the Wnt signaling cascade regulates a variety of pathophysiological conditions in a context-dependent manner and is highly associated with cancers, the identification of Mbd3 as a downstream molecule extends our understanding of Wnt circuits, paving the way for the fine-tuning of Wnt-targeting therapeutics with improved efficacy, and opens new avenues for advancing regenerative therapies targeting neurogenesis-related disorders.

## Materials and methods

### Animals

All animal procedures were approved by the Institutional Animal Care and Use Committee of the Chonnam National University (CNU IACUC-YS-2023-2, protocol no. 202104-746). C57BL/6 pregnant mice were purchased from ORIENT BIO Inc. Mouse embryos and primary neural stem/progenitor cells were obtained from a deceased pregnant mouse after CO_2_ asphyxiation.

### Cell lines

HEK293T reporter cells carrying the chromosomally incorporated TOPflash gene were grown in Dulbecco’s modified Eagle medium (DMEM; Gibco) with 10% fetal bovine serum (Gibco), and 1% penicillin–streptomycin (Gibco) (hereinafter full DMEM) in a humidified atmosphere of 5% CO_2_ at 37 °C. To produce conditioned media (CM), L cells and Wnt3a-producing L cells were cultured in full DMEM. When the cells reach 70% confluence, we provided serum starved condition for 12 h. The media were separately collected and passed through a 0.2-µm filter, generating L-cell-conditioned medium (LCM) and Wnt3a-conditioned medium (Wnt3aCM).

### Primary cell culture

NPCs were isolated from the E11.5 mouse embryo’s cortex in Hanks’ balanced salt solution (Invitrogen) and cultured as described previously^31^. To maintain stem cell characteristics, NPCs were cultured in DMEM/F12 (Gibco) with N2/B27 supplement (Invitrogen) (1:1) supplemented medium containing 10 ng/ml basic fibroblast growth factor (bFGF). To induce conditional differentiation, NPCs were cultured in a medium with withdrawn bFGF and supplied treatments with recombinant Wnt3a, Dkk1 (single and combined) and Wnt5a for 2 days (2-day in vitro (2DIV) differentiation).

### TOPFlash and FOPFlash activity reporter assay

2DIV TOPFlash and FOPFlash (negative control) cells respectively carrying TCF reporter plasmid and mutated TCF binding sites were generated by transfecting the cells with corresponding TCF/LEF vector (TCF/LEF Reporter Kit, BPS Bioscience) following the manufacturer’s protocol, using Lipofectamine Transfection Reagent 3000 (Invitrogen), OptiMEM medium (Gibco). The dual-luciferase reporter assay system (Promega) was used to detect luciferase activity following the manufacturer’s instructions. Luciferase signals were measured using the SpectraMax L Microplate Reader (Molecular Devices LLC).

### Transfection of small interfering RNAs (siRNAs)

To deplete specific target genes, GSK3β siRNA (sc-35525, Santa Cruz Biotechnology), Mbd3 siRNA (sc-35868, Santa Cruz Biotechnology) and HDAC1 siRNA (sc-29344, Santa Cruz Biotechnology) were used and tested against a control siRNA construct (sc-37007). Transfections of siRNAs at a final concentration of 80 nM to NPCs were performed in 60-mm dishes at 70% confluence using Lipofectamine following the manufacturer’s instructions in a final volume of 2 ml OptiMEM. Transfected cells were then maintained in full NPC culture medium, followed by conditional differentiation induction.

### Immunocytochemistry

Cells grown on coverslips were fixed with 4% paraformaldehyde for 10 min, permeabilized with 0.2% Triton X-100, incubated with blocking solution containing 5% bovine serum albumin in phosphate-buffered saline for 30 min and incubated with primary antibodies overnight at 4 °C. After phosphate-buffered saline washing, cells were incubated with secondary antibodies at room temperature for 1 h and counterstained with 4′,6-diamidino-2-phenylindole (DAPI). Images were obtained using a fluorescent confocal microscope (K1 Fluo, Nanoscope Systems). Values obtained from at least three independent experiments were averaged and reported as means ± s.d. The two-tailed Student’s *t*-test was used to compare two experimental groups. To compare more than three experimental groups, one-way analysis of variance (ANOVA) was applied.

### Immunoprecipitation (IP), in vivo ubiquitination assay and immunoblotting

Cells were lysed with IP buffer (50 mM Tris–HCl, pH 7.4, 150 mM NaCl, 1 M NaF, 100 mM EDTA, 0.5% Triton X-100, 1% NP-40, 5% glycerol, 1 mM dithiothreitol and a protease inhibitor cocktail) and then centrifuged at 15,000 rpm at 4 °C for 5 min. The supernatant was collected and precleared with 30 µl of Protein A/G beads (Santa Cruz Biotechnology) for 2 h and then incubated with 4 µg of each specific antibody overnight at 4 °C. Then, immune complexes were washed six times with IP buffer and eluted by boiling for 5 min at 95 °C in SDS sample buffer, then separated on 10% SDS–PAGE and immunoblotted as previously described^32^. After blocking the membranes with 5% skim milk, membranes were sequentially incubated with corresponding primary antibodies, followed with HRP-conjugated secondary antibodies (anti-mouse or anti-rabbit 1:10,000). Protein bands were detected using enhanced chemiluminescence (ECL) reagent.

### In vivo cross-linking and ChIP–qPCR

For the chromatin immunoprecipitation (ChIP) assay, 36–38% formaldehyde was added to the overlaying media to achieve a final concentration of 1% formaldehyde, followed by incubation on a shaker at room temperature for 10 min. Cross-linking was quenched by adding glycine to a final concentration of 0.125 M, with an additional incubation on a shaker at room temperature for 5 min. Chromatin was then sonicated using Pico Bioruptor (Diagenode) for 5 cycles of 30 s (15 s on; 15 s off) to fragment DNA to 200–1,000 bp (Supplementary Fig. 4). Sheared chromatin was cleared by centrifugation at 16,000*g* for 10 min at 4 °C. The collected supernatant was immunoprecipitated overnight with rabbit anti-IgG, anti-MBD3, anti-HDAC1 and anti-MTA1 antibodies at 4 °C, followed by incubation with 50 μl of magnetic Protein A/G Dynabeads (MilliporeSigma). Next, DNA was eluted from immunoprecipitates, cross-links were reversed, and the DNA was purified. The abundance of target sequences was analyzed using quantitative PCR (qPCR; CFX Duet, Bio-Rad) and normalized as fold enrichment relative to input chromatin. The primers used for ChIP–qPCR are listed in Supplementary Table 1.

### Fractionation assay

Nuclear and cytoplasmic fractions of mouse NPCs were isolated following a previously published protocol^33^. In brief, cells were completely lysed in 300 µl of hypotonic lysis buffer, incubated on ice for 10 min and then centrifuged at 200*g* for 2 min. The supernatant, containing cytoplasmic fraction, was transferred to a prechilled tube and kept on ice. The remaining pellet was washed three times by adding 300 µl of hypotonic lysis buffer, gently suspending and centrifuging at 200*g* for 2 min, followed by a complete decant of the supernatant. This washing step was repeated twice more, after which the resultant pellet was resuspended and sonicated using VCX130 vibra-cells (Sonics) for 15 s at 60% amplitude in 300 µl of nuclear lysis buffer, resulting in nuclear fraction. Cytoplasmic and nuclear fractions then underwent centrifugation for 15 min at 20,000*g*, and the supernatants were collected for western blot analysis.

### Real-time PCR (RT-PCR)

2DIV differentiated NPCs from each treatment group were collected for total RNA extraction using TRIzol reagent (Invitrogen). cDNA synthesis was performed using the SuperScript III qRT-PCR kit (Invitrogen). RT-PCR was carried out with specific primers using the 6331 Nexus Gradient MasterCycler Thermal Cycler (Eppendorf) and THUNDERBIRD Next SYBR qPCR Mix (Toyobo). The reaction conditions included an initial denaturation at 95 °C for 60 s, followed by 40 cycles of denaturation at 95 °C for 15 s, annealing at 55 °C for 15 s and extension at 72 °C for 60 s. The resulting samples were confirmed by electrophoresis to assess mRNA expression levels. Primer sets for RT-PCR are listed in Supplementary Table 2.

### Antibodies and reagents

Antibodies used in this study were anti-β-catenin (Cell Signaling), anti-Mbd3 (Cell Signaling Technology), anti-α-tubulin (Abcam), anti-CD133 (Sigma-Aldrich), anti-Ki67 (Sigma-Aldrich), anti-TUJ1 (Abcam), anti-MAP2 (Abcam), anti-Nestin (Abcam), anti-IgG, anti-HDAC1 (Abcam), anti-Myc (Abcam), anti-V5 (Abcam), anti-MTA1 (Abcam), anti-GAPDH (Abcam) and anti-histone H3 (Abcam). Secondary antibodies were HRP-conjugated anti-rabbit and anti-mouse, anti-rabbit Alexa Fluor 488-, anti-mouse Alexa Fluor 488-, anti-rabbit Alexa Fluor 555- and anti-mouse Alexa Fluor 555-conjugated IgG (Molecular Probes). DAPI was from Millipore-Sigma. Recombinant (rc)Wnt3a, rcDKK1 and rcWnt5a were purchased from R&D Systems. Proteasome inhibitor MG132, CHIR99021 (CHIR) and cycloheximide (CHX) were purchased from Cayman Chemical. bFGF was from PeproTech. The protease inhibitor cocktail was purchased from Roche Applied Science. The ECL (Peirce ECL western blotting substrate) kit was from Thermo Fisher Scientific. RNase A, Proteinase K and phenol–chloroform–isoamyl alcohol were purchased from Sigma-Aldrich.

### Prediction of phosphorylation sites

Mbd3 protein sequences from the UniProt database (https://www.uniprot.org/) were used as query sequences for phosphorylation sites prediction using Group-based Prediction System (GPS) version 6.0 (https://gps.biocuckoo.cn/) as previously described^34^.

### Quantification of ubiquitination IP blots

Ubiquitination IP blot quantification was performed using ImageJ software. Each band on the IP blot was manually selected to measure integrated intensity, with background correction applied to subtract nonspecific signals.

For normalization, the quantified ubiquitination signal was normalized to the corresponding control group (the first experimental group) within the same blot to account for variations in protein loading and transfer efficiency. Similarly, the relative intensity of the α-tubulin band was calculated for each group. The final quantification value used for graph plotting was obtained by dividing the relative ubiquitination level by the corresponding α-tubulin intensity, with the control group set to 1.0 (arbitrary units). Data plotted were obtained from at least three independent blot replicates.

### Statistical analysis

Each experiment was performed at least three times (*n* ≥ 3). All statistical analyses were performed using Excel statistical tools or Prism 9 (GraphPad Software). Where differences between treatment groups were experimentally hypothesized, the data were assessed for normality and variance homogeneity. For comparisons between two groups, an unpaired Student’s *t*-test was used. For comparisons among multiple groups, one-way ANOVA followed by Tukey’s multiple-comparison test was conducted. In all statistical analyses, *P* < 0.05 was considered significant (*P < 0.05, **P < 0.01, ***P < 0.001).

## Results

### Canonical Wnt signaling promotes Mbd3 protein stability

**Fig. 1:**
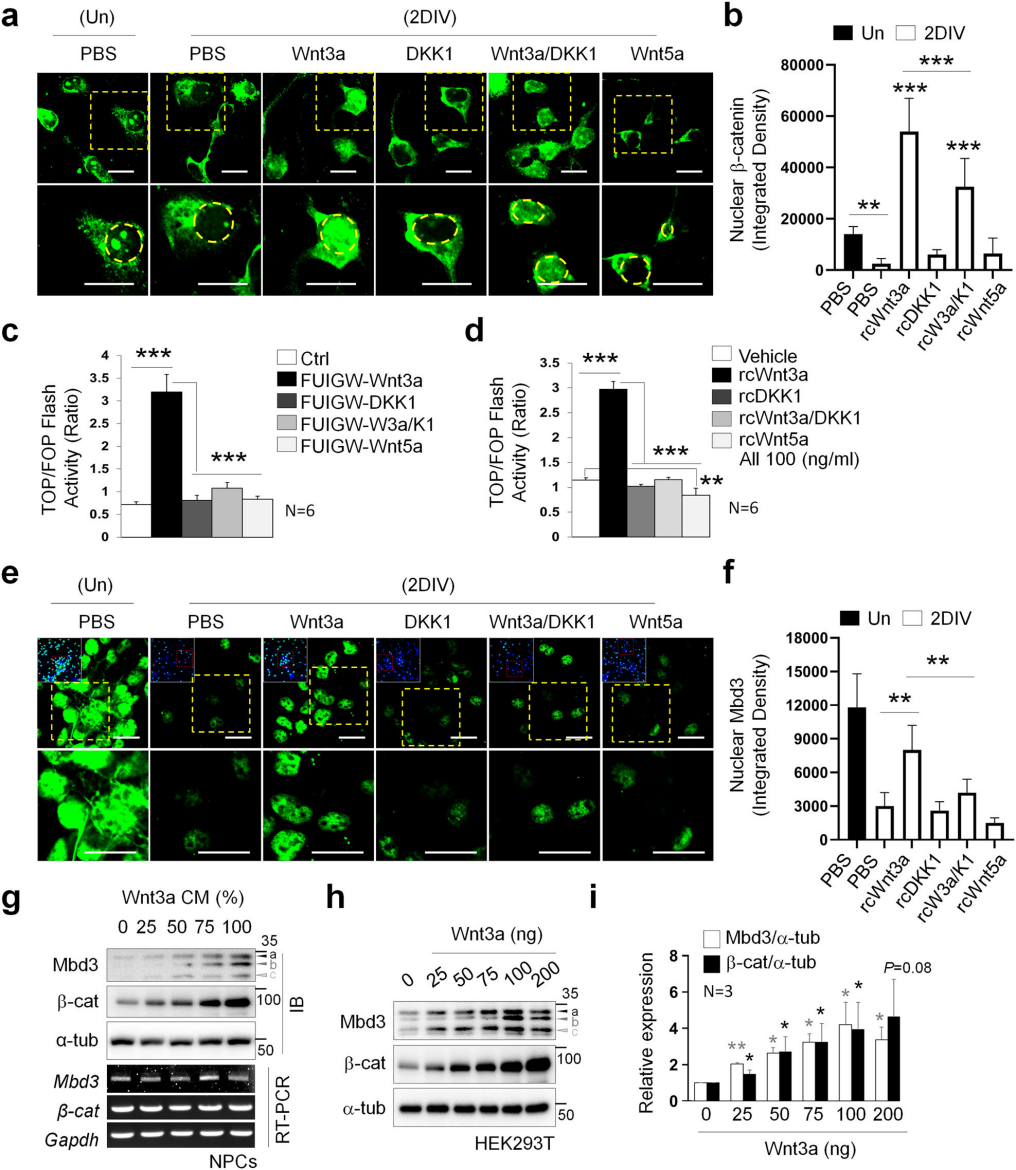
Canonical Wnt signaling promotes Mbd3 stabilization. **a** Immunostaining detecting nuclear localization of β-catenin (green) in undifferentiated (Un) and 2DIV differentiated NPCs in different states of Wnt signaling (*N* = 3). Scale bar, 50 µm. **b** Quantification of nuclear β-catenin intensity in undifferentiated and 2DIV differentiated cells in different states of Wnt signaling (*N* = 3). **c** A plot of the TOP/FOP Flash assay indicating the β-catenin level through luciferase activity in differentiated NPCs overexpressing Wnt activators (Wnt3a (canonical signaling), Wnt5a (noncanonical signaling)) or inhibitor (DKK1) (*N* = 6). **d** A plot of the TOP/FOP Flash assay indicating the β-catenin level through luciferase activity in differentiated NPCs under the supplements of the indicated rcWnt signaling stimuli (*N* = 6). **e** Immunostaining detecting nuclear localization of Mbd3 (green) in undifferentiated and 2DIV differentiated NPCs in different states of Wnt signaling. Scale bar, 50 µm. **f** Quantification of Mbd3 expression level in undifferentiated and 2DIV differentiated NPCs in different states of Wnt signaling (*N* = 3). **g** The expression of β-catenin and Mbd3 at the protein level (by immunoblot (IB), top) and mRNA level (by RT-PCR, bottom) in 2DIV differentiated NPCs treated with varying doses of Wnt3aCM (*N* = 3). Labels a–c denote Mbd3 isoforms. **h** Western blot showing β-catenin and Mbd3 expression level in HEK293T cells with dose-dependent supplement of Wnt3a protein (*N* = 3). Labels a–c denote Mbd3 isoforms. **i** Quantification of the relative expression of β-catenin and Mbd3 of **h** In all cases, data are presented as mean ± s.d.; one-way ANOVA was performed to calculate significance (*P < 0.05, **P < 0.01, ***P < 0.001).

Our previous study explored the close association between Mbd3 and the neurogenesis potential of NPCs^30^. In addition, emerging evidence has identified the Wnt signaling pathway as a factor promoting neural lineage commitment in embryonic stem cells^21^. To investigate whether Mbd3 is involved in Wnt signaling-induced neurogenesis, we examined its expression pattern in differentiated NPCs under various Wnt signaling contexts, targeting both the canonical and the noncanonical branches. Accordingly, we first determined the expression pattern of β-catenin—a crucial nuclear effector of the canonical Wnt pathway—as a reference protein. Immunocytochemistry (ICC) analysis revealed that active β-catenin, which accumulates in the nucleus and is highly expressed in undifferentiated NPCs, was significantly reduced following 2DIV differentiation (Fig. 1a, b). Differentiation in the presence of recombinant Wnt3a (rcWnt3a), a prototype of canonical Wnt ligand, meanwhile, substantially increased β-catenin nuclear localization. By contrast, treatment with Dickkopf-1 (DKK1), a Wnt signaling inhibitor, markedly decreased active β-catenin levels. When combined with Wnt3a, Dkk1 exhibited an antagonistic effect, leading to reduced nuclear β-catenin accumulation compared with treatment with rcWnt3a alone. Meanwhile, untreated and rcWnt5a-treated 2DIV groups showed no significant difference in the level of nuclear-localized β-catenin. Given that Wnt5a is an upstream effector of noncanonical Wnt signaling, this result suggests the independence of β-catenin levels from the noncanonical branch of the Wnt pathway.

We next used the TOPFlash and FOPFlash reporter system to further characterize β-catenin expression in the neural differentiation of NPCs under the regulation of the Wnt cascade. First, we induced the overexpression of the examined Wnt modulatory protein by transfecting NPCs with Wnt3a, DKK1 and Wnt5a constructs cloned into FUIGW plasmid backbones and evaluated luciferase activity of the generated cells after differentiation (Fig. 1c). Overexpression of Wnt3a substantially increased luciferase activity compared with the control, indicating that Wnt3a significantly promotes active β-catenin levels. This effect, however, was counteracted by either DKK1 or Wnt3a–DKK1 overexpression, in which the combined overexpression resulted in higher β-catenin level, demonstrating that DKK1 repressed β-catenin-dependent Wnt signaling by antagonizing Wnt3a. Concordant with the ICC result, we observed that gain of function of Wnt5a could not promote β-catenin accumulation in 2DIV cells. To exclude any potential factors that could affect the expression of the transduced genes, we validated these observations by analyzing TOP and FOP Flash activity of 2DIV cells cultured in the presence of exogenous recombinant regulatory proteins and observed effects comparable to those recorded in the transgenic cells (Fig. 1d).

Next, investigating the Mbd3 expression pattern through ICC, we found an intense accumulation in the nuclear regions of NPCs (Fig. 1e, f). After differentiation induction, Mbd3 nuclear localization was substantially reduced. This translocation could be substantially promoted or suppressed, respectively, by activating or inhibiting the Wnt signaling cascades through the supplementation with rcWnt3a ligand or rcDKK1. Interestingly, Mbd3 levels in the DKK1–Wnt3a combinatory treatment were significantly decreased compared with the rcWnt3a-treated group, revealing that Mbd3 expression could be regulated by β-catenin modulators. Furthermore, Wnt5a noncanonical ligand could not affect Mbd3 nuclear levels, suggesting similarities in the expression pattern of Mbd3 and active β-catenin. To further validate the localization of both proteins, we examined their levels in nuclear and cytoplasmic fractions of NPCs (Supplementary Fig. 1). Consistent with our ICC results, β-catenin and Mbd3 showed similar expression patterns in the nuclear fractions, in which the significant reduction after differentiation could be rescued following rcWnt3a treatment. Interestingly, cytoplasmic β-catenin exhibited similar expression patterns, albeit to a lesser extent. Meanwhile, Mbd3 was expressed at low levels in the cytoplasmic fractions, with less pronounced changes.

To gain insights into the impact of canonical Wnt signaling activation on Mbd3 protein, we induced differentiation of NPCs in dose-dependent Wnt3aCM. Examining protein expression of 2DIV cells by western blot (Fig. 1g), we found that Mbd3 levels were positively correlated with Wnt3a concentrations, indicating the possible regulatory role of β-catenin signaling in Mbd3 stability. The corresponding mRNA levels, demonstrated by RT-PCR results, revealed no significant differences across the dose-dependent treatments, concordant with our previous finding^30^ that these proteins are regulated primarily at the protein level. Further validating the effect of Wnt3a using recombinant protein, we also observed dose-responsive upregulations in the expression levels of β-catenin and Mbd3 (Fig. 1h, i). Together, these results suggest canonical Wnt signaling cascades as a synergistic regulator for Mbd3 stability during neurogenesis, in which enhancement of the pathway through the presence of activating ligands increases the level of Mbd3.

### Canonical Wnt signaling inhibits Mbd3 destabilization

**Fig. 2:**
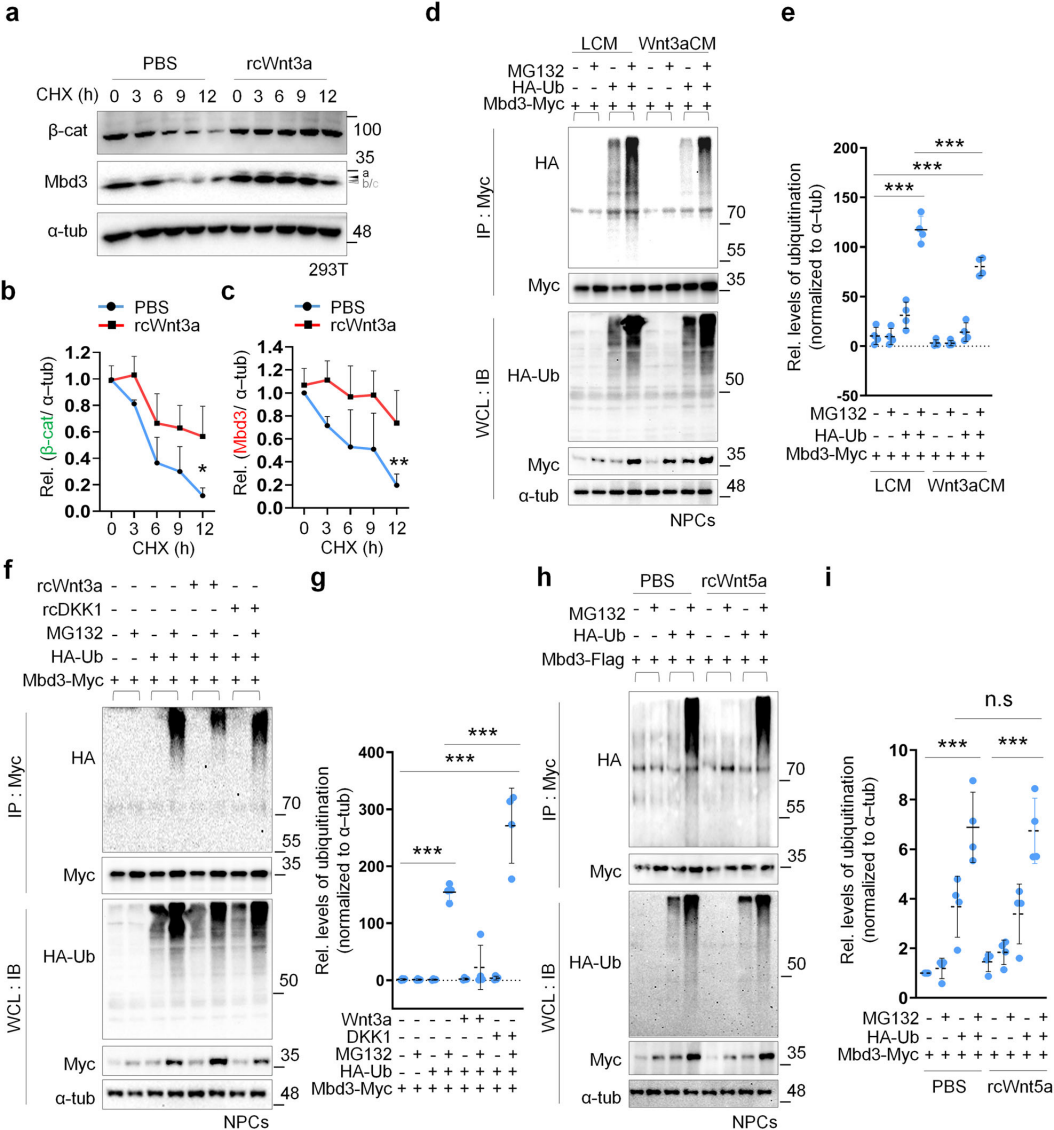
Canonical Wnt signaling inhibits Mbd3 stabilization. **a** Immunoblot (IB) analysis of β-catenin and Mbd3 in HEK293T cells treated time-dependently with CHX, followed by activation of canonical Wnt signaling through rcWnt3a supplementation. Labels a–c denote Mbd3 isoforms. **b**, **c** Corresponding quantification of the relative expression level of β-catenin (**b**) and Mbd3 (**c**) in the indicated conditions. **d** IP of HA-tagged ubiquitin (HA-Ub) in NPCs expressing full-length Myc-tagged Mbd3 (Myc-Mbd3) in LCM and Wnt3aCM (*N* = 3). Levels of each protein in the whole-cell lysate (WCL) are shown with western blot. IP was demonstrated using an anti-Myc antibody. **e** The relative level of Mbd3 ubiquitination in LCM and Wnt3aCM. **f** Myc-Mbd3 and HA-Ub expression vectors were transfected to NPCs and subjected to differentiation in Wnt3a or DKK1 treatment with or without MG132 (1 day later), then co-IP with anti-Myc antibody (*N* > 3). **g** The relative level of Mbd3 ubiquitination in Wnt3a and DKK1 conditions. **h** IP of HA**-**Ub in NPCs carrying control vector (FUIGW) or Wnt5a-overexpressing vector (FUIGW-Wnt5a) (*N* = 3). **i** The relative level of Mbd3 ubiquitination in negative control and FUIGW-Wnt5a conditions. In all cases, data are presented as mean ± s.d.; one-way ANOVA was performed to calculate significance (*P < 0.05, **P < 0.01, ***P < 0.001).

To gain comprehensive insight into the regulatory function of Wnt signaling on Mbd3 protein, we investigated whether this pathway is involved in the regulation of Mbd3 stability. Our previous data indicated the differential dynamics of Mbd3 and β-catenin in protein expression, rather than mRNA expression, urging us to examine their protein levels over time in the presence of Wnt3a. Following CHX treatment, which blocks new protein synthesis, β-catenin and Mbd3 in HEK293T cells were gradually degraded over time; however, exogenous Wnt3a rescued and promoted their stability over the investigated period (Fig. 2a–c). This suggested that canonical Wnt signaling activation could potentially arrest the destruction of Mbd3 in differentiated NPCs.

Our previous study explored the destabilization of Mbd3 by ubiquitination during neural differentiation^30^. Based on this discovery, we further investigated the role of Wnt signaling in Mbd3 ubiquitination by performing IP with Myc-tagged Mbd3 and HA-ubiquitin expressed in 2DIV cells of NPCs. In LCM, we captured specific interactions between ubiquitin and Mbd3 in the group treated with proteasome inhibitor MG132, revealing the occurrence of ubiquitination in endogenous Mbd3 (Fig. 2d, e). Wnt3aCM, meanwhile, resulted in a lower level of ubiquitin–Mbd3 interaction, suggesting the function of canonical Wnt signaling activators in attenuating Mbd3 ubiquitination. Interestingly, we observed similar results when directly supplying treatment proteins to the culture medium of 2DIV cells. Under MG132 conditions, the absence of Wnt3a resulted in Mdb3 polyubiquitination while its supplementation rescued Mdb3 from this destabilization event (Fig. 2f, g). Furthermore, deactivation of Wnt–β-catenin signaling through the addition of DKK1 triggered the ubiquitination of endogenous Mbd3 within differentiated cells, showing the effect of canonical Wnt cascades regulators on Mbd3 stability. At the same time, to assess whether noncanonical Wnt signaling regulates Mbd3 stability, we added the Wnt5a prototypic ligand to differentiated NPCs. After proteasome inhibition, we observed that the level of polyubiquitinated Mbd3 accumulated in the treated cells was comparable to that in the control group (Fig. 2h, i), suggesting that noncanonical Wnt signaling does not exert suppressive effects on Mbd3 destabilization. Together, our findings validate the potential of canonical Wnt signaling in inhibiting the destruction of Mbd3, thereby promoting its stability during neurogenesis.

### GSK3β inhibition promotes Mbd3 protein stability

**Fig. 3:**
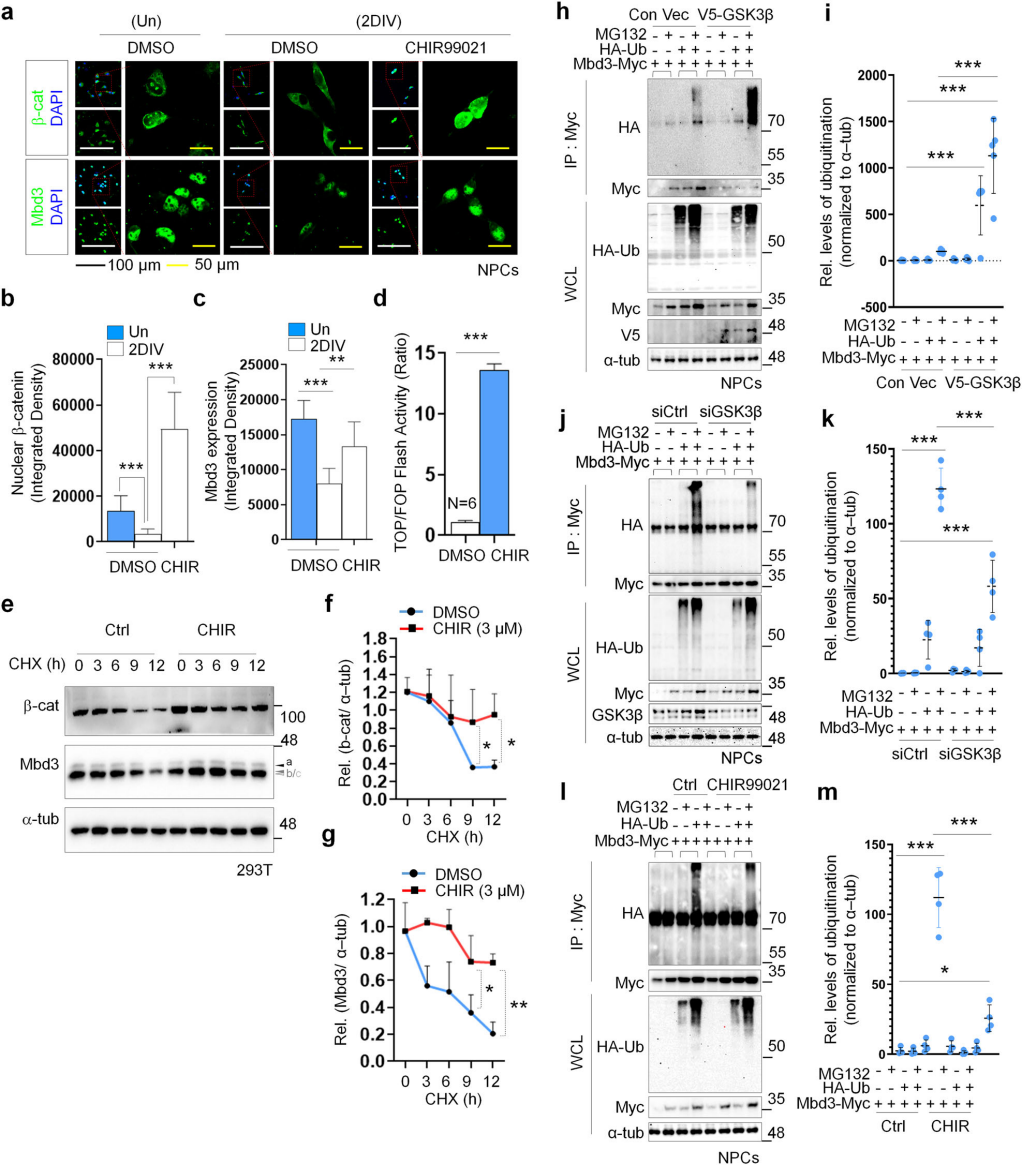
GSK3β inhibition promotes Mbd3 protein stability. **a** Immunostaining detecting β-catenin and Mbd3 enrichment in undifferentiated NPCs and 2DIV differentiated NPCs treated with GSK3β inhibitor (CHIR). Nuclear staining is shown by DAPI (blue). Scale bars, 100 µm (black) and 50 µm (yellow). **b**, **c** Quantification of protein levels of β-catenin (**b**) and Mbd3 (**c**) in undifferentiated and 2DIV differentiated NPCs. **d** A plot of TOP/FOP Flash assay indicating β-catenin levels through luciferase activity in CHIR-treated differentiated NPCs. **e** Time-dependent levels of β-catenin and Mbd3 protein in HEK293T cells following CHX and with or without CHIR treatment (*N* = 3). Labels a–c denote Mbd3 isoforms. **f**, **g** Corresponding quantification of protein levels of β-catenin (**f**) and Mbd3 (**g**). **h** IP of HA-Ub in the NPCs expressing full-length Myc-Mbd3 with control or V5 tagged GSK3β (V5-GSK3β) expression vector (*N* = 3). IP was demonstrated using an anti-Myc antibody. Overexpression of GSK3β was shown through anti-V5 antibody. **i** Corresponding relative level of Mbd3 ubiquitination in GSK3β overexpression. **j** Myc-Mbd3 and HA-Ub expression vectors were transfected to NPCs carrying control siRNA or GSK3β siRNA and subjected to co-IP with anti-Myc antibody (*N* > 3). **k** Corresponding relative level of Mbd3 ubiquitination in GSK3β silence. **l** IP of HA-Ub in the NPCs expressing full-length Myc-tagged Mbd3 (Myc-Mbd3) with or without CHIR treatment (*N* = 3). **m** Corresponding relative level of Mbd3 ubiquitination in GSK3β overexpression induced by CHIR. In all cases, data are presented as mean ± s.d. Statistical significance was determined using an unpaired two-tailed Student’s *t*-test for **b**–**d** (*P < 0.05, **P < 0.01, ***P < 0.001) and one-way ANOVA for **f**, **g**, **i**, **k** and **m** (*P < 0.05, **P < 0.01, ***P < 0.001).

Activation of the canonical Wnt pathway, indeed, is the inhibition of destruction complex formation whose role is to keep β-catenin levels in check through degradation. In this process, glycogen synthase kinase 3 beta (GSK3β) is critical for β-catenin stability because it functions as the principal kinase marking β-catenin for further ubiquitination and eventually proteasomal degradation^35,36^. Our evidence above demonstrated that the canonical Wnt pathway could also regulate Mbd3 stabilization. Therefore, to elucidate the regulatory function of canonical Wnt signaling on Mbd3, we studied whether GSK3β involves Mbd3 stabilization during neurogenesis. For this purpose, we first inhibited GSK3β by delivering CHIR treatment during differentiation of NPCs, then characterized the nuclear localization of β-catenin and Mbd3 by ICC (Fig. 3a). Interestingly, CHIR not only restored but also significantly enhanced their nuclear expression in 2DIV cells (Fig. 3b, c). Meanwhile, analyzing the luciferase activity of 2DIV cells carrying the reporter plasmid, we found a significant increase in the CHIR-treated group (Fig. 3d), confirming GSK3β’s role in β-catenin stabilization. To determine whether manipulating GSK3β also affects Mbd3 stability, we examined the time-dependent degradation of endogenous Mbd3 and β-catenin in the absence and presence of GSK3β inhibitor (Fig. 3e) and observed reductions in their levels over the examined period (Fig. 3f, g). Significantly, CHIR prolonged their stability, indicating that GSK3β, along with controlling β-catenin degradation, also regulates Mbd3 destabilization.

Next, we characterized the effects of GSK3β on Mbd3 stability during neural differentiation. We overexpressed GSK3β by transfecting NPCs with GSK3β-V5 tagged vector and induced differentiation. Through IP, we captured the conjugation of Mdb3 with ubiquitin while withdrawing proteasomal activity using MG132. Notably, ubiquitinated Mbd3 levels in GSK3β overexpressed cells was drastically higher than those of the control (Fig. 3h, i). At the same time, we examined Mbd3 destabilization in the siRNA-mediated knockdown of GSK3β and observed a reduction in Mbd3 polyubiquitination level compared with the negative control (Fig. 3j, k), supporting the involvement of GSK3β in Mbd3 degradation. This effect was validated when CHIR treatment obstructed the polyubiquitination of Mbd3 compared with the control (Fig. 3l, m).

To uncover the interaction between GSK3β and Mbd3, we utilized the GPS server and identified 33 potential serine or threonine residues in Mbd3 that could be specifically phosphorylated by GSK3β (Supplementary Fig. 2a–c). To assess whether GSK3β and Mbd3 form a complex, we conducted co-IP with V5-GSK3β and HA-Mbd3 expressed in 2DIV NPCs and observed interactions under physiological condition (Supplementary Fig. 2d), supporting the GSK3β–Mbd3 interaction. Together, these findings indicate GSK3β as a factor contributing to inducing Mbd3 degradation through polyubiquitin-dependent proteasomal pathway and highlight the potential role of GSK3β inhibition in promoting Mbd3 protein stability.

### Canonical Wnt signaling and GSK3β inhibition maintains stemness of NPCs

**Fig. 4:**
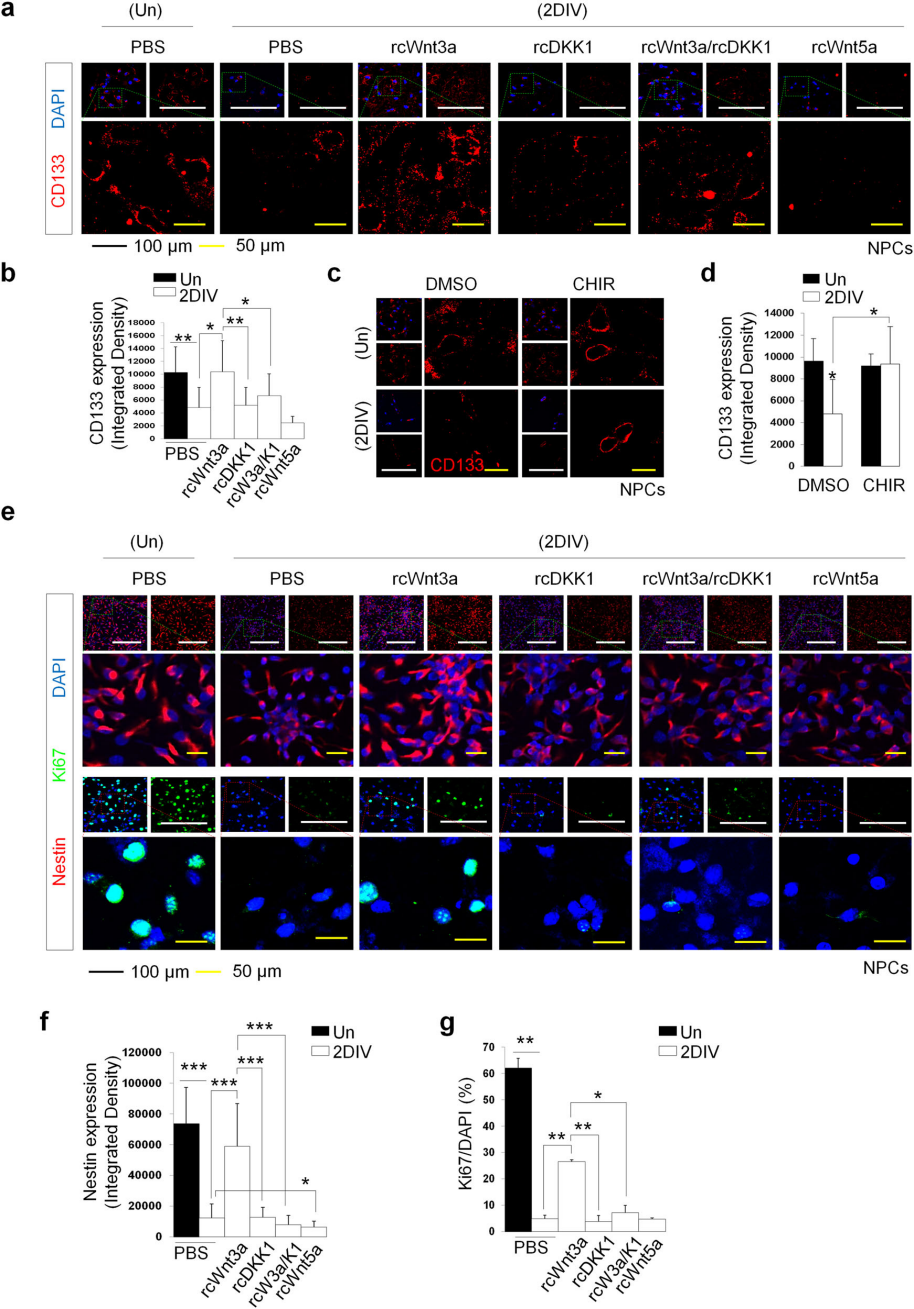
Activation of canonical Wnt signaling maintains stemness of NPCs. **a** Immunostaining to detect the expression of CD133 stemness biomarkers (red) in undifferentiated and 2DIV differentiated NPCs under different stimuli on Wnt signaling. Scale bars, 100 µm (black) and 50 µm (yellow). **b** Corresponding quantification of CD133 expression level of undifferentiated and 2DIV differentiated NPCs in each indicated condition. **c** Immunostaining detecting the expression of CD133 (red) in undifferentiated NPCs and 2DIV differentiated NPCs with CHIR treatment. Scale bars, 100 µm (black) and 50 µm (yellow). **d** Corresponding quantification of CD133 expression level in undifferentiated and 2DIV differentiated NPCs with CHIR supplement. **e** Immunostaining detecting the expression of Nestin neural stem cell marker (red) and Ki67 proliferative marker (green) in undifferentiated and 2DIV differentiated NPCs under different stimuli on Wnt signaling. Scale bars, 100 µm (black) and 50 µm (yellow). **f**, **g** Corresponding quantification of Nestin (**f**) and Ki67 (**g**) expression level of undifferentiated and 2DIV differentiated NPCs in each indicated condition. In all cases, data are presented as mean ± s.d. Statistical significance was determined using an unpaired two-tailed Student’s *t*-test for **d** (*P < 0.05, **P < 0.01, ***P < 0.001) and one-way ANOVA for **b**, **f** and **g** (*P < 0.05, **P < 0.01, ***P < 0.001).

Our experiment highlights the critical role of canonical Wnt signaling and GSK3β inhibition in retaining Mbd3 protein stability. In the previous study, we explored that Mbd3 overexpression significantly blocks NPC differentiation^30^. Furthermore, Mbd3 degradation has been shown to compromise stem-like properties, as indicated by reduced CD133 (promin-1) expression both in vitro and in vivo^37^. Therefore, we investigated the influence of canonical Wnt signaling and GSK3β inhibition on the stemness of NPCs. Through ICC analysis, we found that the CD133-positive population was significantly reduced after NPC differentiation (Fig. 4a, b). Notably, we found that the supplementation of prototypic canonical Wnt signaling ligand Wnt3a substantially rescued CD133 expression in 2DIV. By contrast, this effect was counteracted by the Wnt–β-catenin pathway inhibitor DKK1. Moreover, no enhancement in stem-like property biomarker expression was recorded in the Wnt5a-treated group. Concurrently, to study the function of GSK3β in maintaining stemness, we compared the expression level of CD133 cell surface protein in NPCs and CHIR-treated 2DIV cells (Fig. 4c). We found that GSK3β inhibition remarkably elevated CD133 levels in differentiated cells (Fig. 4d), indicating a positive correlation between progenitor cell stemness and GSK3β suppression. In addition, ICC analysis targeting neural stem cell marker Nestin and proliferative marker Ki67 demonstrated a significant increase in the expression of these proteins in Wnt3a-treated 2DIV cells compared with the control (Fig. 4e–g). By contrast, the supplementation with DKK1 reduced the level of these proteins, reflecting the regulatory role of canonical Wnt cascades in the acquisition of NPCs stemness. No promoting effect on Nestin and Ki67 expression was observed in the rcWnt5a-treated group, revealing that activation of the noncanonical Wnt pathway via Wnt5a is not associated with the stem-like properties of NPCs. Together, our results validate the significance of Wnt3a and GSK3β inhibition (that is, the activation of canonical Wnt cascades) in retaining the stemness of NPCs.

### Canonical Wnt signaling and GSK3β inhibition abrogates neuronal differentiation of NPCs

**Fig. 5:**
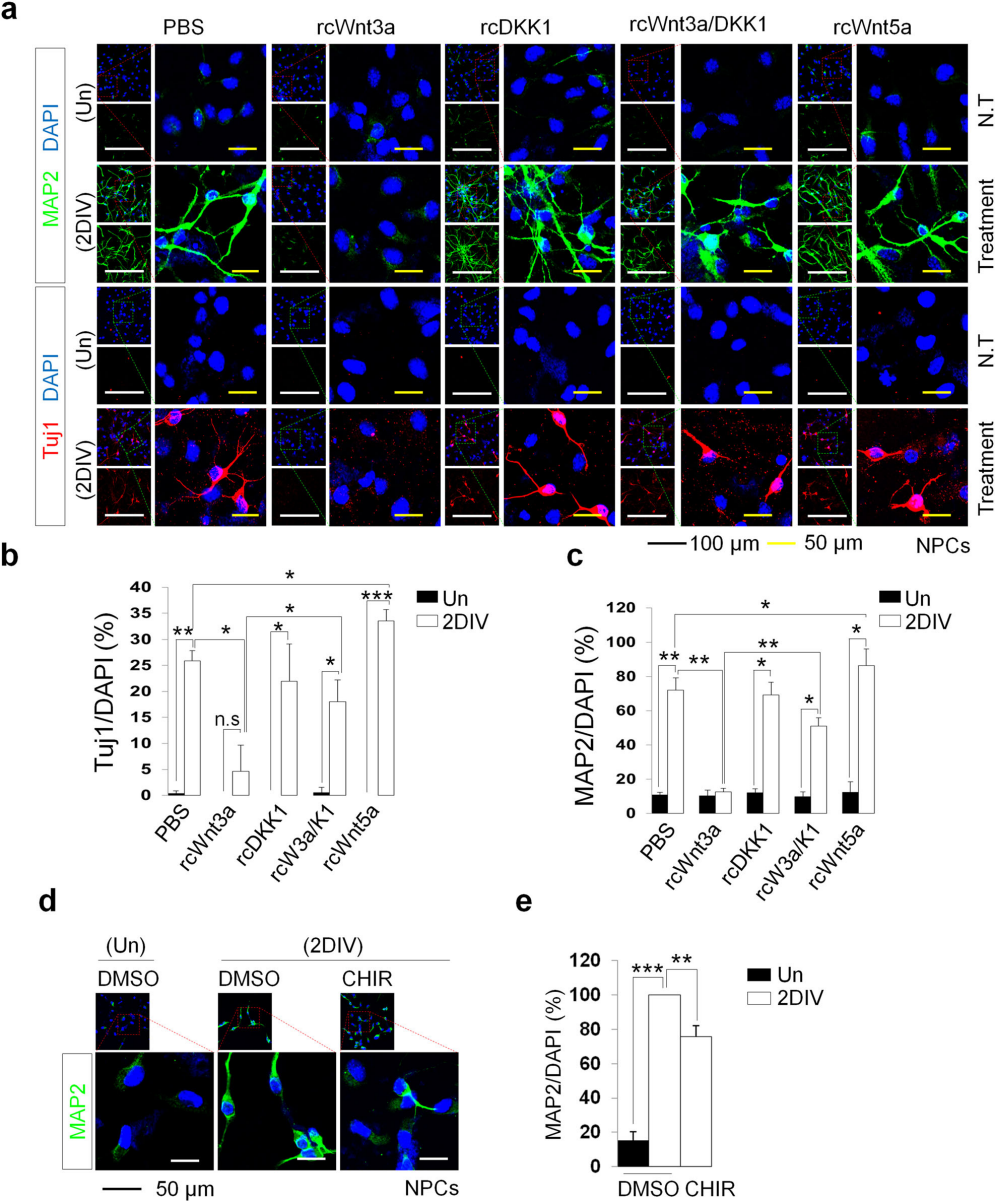
Canonical Wnt signaling cascade activation abrogates neuronal differentiation of NPCs. **a** Immunostaining to detect the expression of Tuj1 (red) and MAP2 (green) biomarkers for early and mature neurons in undifferentiated and 2DIV differentiated NPCs under different stimuli on Wnt signaling. Scale bars, 100 µm (black) and 50 µm (yellow). **b**, **c** Corresponding quantification of Tuj1-expressing (**b**) and MAP2-expressing (**c**) cells of undifferentiated and differentiated NPCs under each indicated condition. **d** Immunostaining detecting the expression of MAP2 (green) in undifferentiated NPCs and 2DIV differentiated NPCs treated with CHIR. Scale bar, 50 µm (black). **e** Corresponding quantification of MAP2 in undifferentiated and differentiated NPCs with CHIR supplement. In all cases, data are presented as mean ± s.d.; one-way ANOVA was performed to calculate the significance (*P < 0.05, **P < 0.01, ***P < 0.001).

Having shown the effects of canonical Wnt signaling activation in stemness maintenance of NPCs, we next elucidated their roles in neuronal differentiation process. From ICC analysis, we observed a significant increase in the population of early and mature neurons (Tuj1- and MAP2-positive cells, respectively) after NPC differentiation (Fig. 5a–c). However, under Wnt3a stimulation, neurogenesis was greatly suppressed, evident from the drastically decreased number of early and mature neurons (by approximately fourfold and sevenfold, respectively, compared with the control group). In addition, the inhibition of the Wnt signaling pathway mediated by DKK1 significantly enhanced neurogenesis. In the combined treatment, DKK1 antagonized Wnt3a in activating Wnt–β-catenin signaling, resulting in a higher number of Tuj1- and MAP2-expressing cells. Furthermore, when NPCs were differentiated in the Wnt5a condition, we observed a substantial increase in early and mature neurons, compared with the control group, revealing that noncanonical Wnt signaling could not suppress the neuronal differentiation of NPCs. As the activation of canonical Wnt signaling cascade induced by Wnt3a was shown to abrogate neurogenesis from NPCs, we validated this observation by examining the effect of GSK3β that plays a key role in this pathway. We found that inhibition of GSK3β via CHIR significantly suppressed NPC differentiation, evident from the decreased proportion of mature neurons in 2DIV group compared with the control (Fig. 5d, e). These results, therefore, emphasize the crucial role of active canonical Wnt signaling in abrogating the neurogenesis of NPCs.

### Canonical Wnt signaling promotes enrichment of Mbd3–NuRD complex on neurogenesis-associated genes

**Fig. 6:**
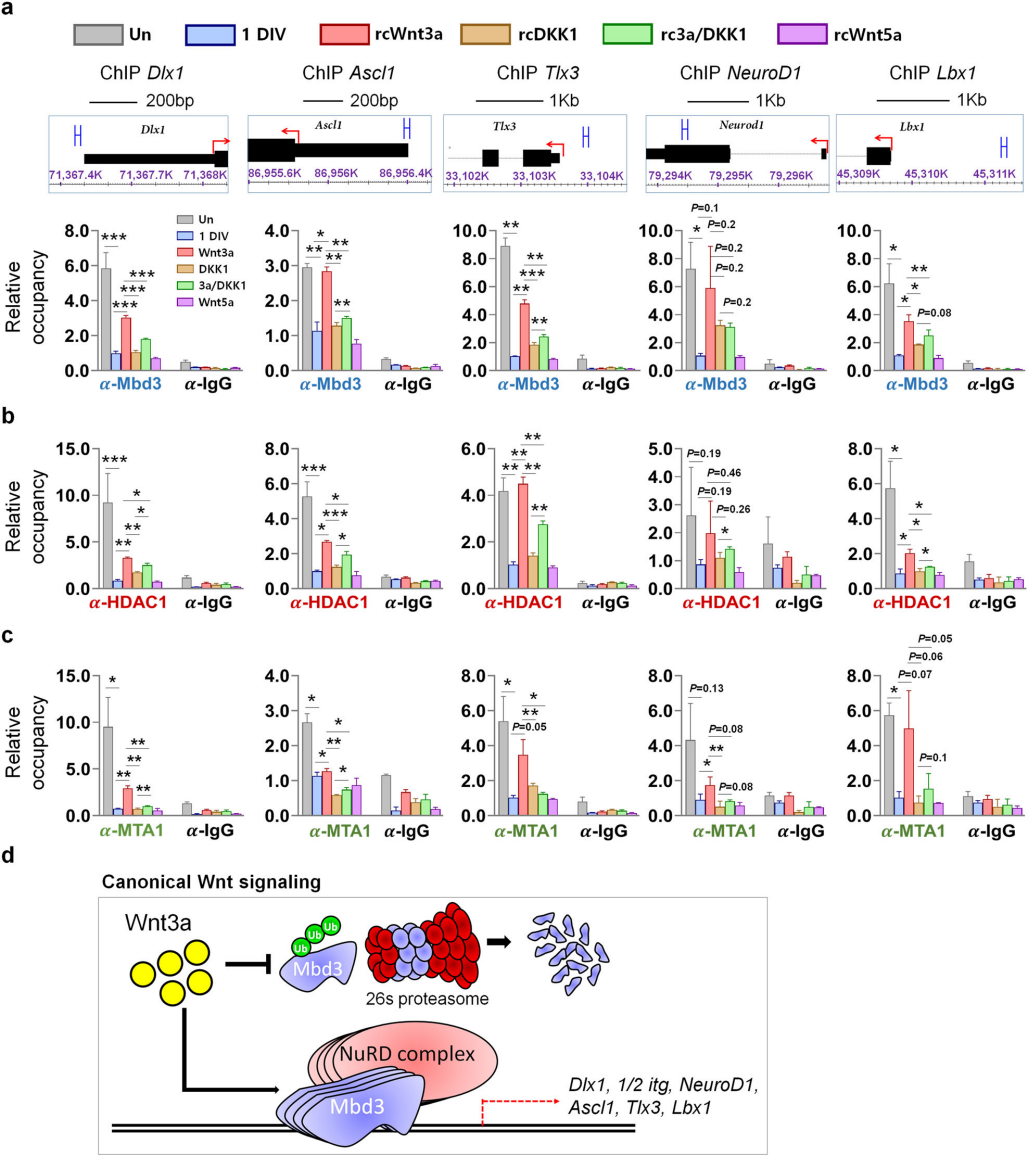
Wnt signaling states regulate transcription of neuronal genes through the recruitment of Mbd3–NuRD complex components during differentiation of NPCs. **a**–**c** ChIP–qPCR analysis of Mbd3 (**a**), HDAC1 (**b**) and MTA1 (**c**) occupancy at Mbd3-binding loci in undifferentiated and differentiated cells in different stimuli for the Wnt signaling cascade (*N* = 3). Data are presented as mean ± s.d.; one-way ANOVA was performed to calculate the significance (*P < 0.05, **P < 0.01, ***P < 0.001). **d** A model of the regulatory role of canonical Wnt signaling on cell-fate determination during neurogenesis, in which Mbd3–NuRD functions downstream to suppress the transcription of neural differentiation genes, thereby maintaining the stem cell pool.

We previously identified the epigenetic regulatory role of Mbd3 in NPCs, which operates by facilitating the recruitment of NuRD complex components to neurogenesis-related genes, thereby suppressing their transcription^30^. Given that active canonical Wnt signaling abrogates the neural differentiation potential of NPCs, we next investigated whether the Mbd3–NuRD complex functions in this regulation. For this purpose, we first performed ChIP–qPCR with anti-Mbd3 antibody to capture alterations in Mbd3 enrichment on the five genes inducing neuronal program (*Dlx1*, *Ascl1*, *Tlx3*, *NeuroD1* and *Lbx1*) that have been discovered as Mbd3 interaction partners in our prior studies^30,37^. We observed that Mbd3 occupancy on the examined neuronal gene loci was remarkably high in undifferentiated NPCs, then substantially reduced after 1-day differentiation in vitro (1DIV) (by 5.9-, 2.6-, 8.9-, 6.8- and 5.8-fold, respectively, in *Dlx1*, *Ascl1*, *Tlx3*, *NeuroD1* and *Lbx1* genes) (Fig. 6a). In addition, compared with the control, Wnt3a stimulation substantially enhanced the binding of Mbd3 on these genes, whereas DKK1 treatment counteracted this effect in both single and cotreatment with Wnt3a. Moreover, supplementation with Wnt5a ligands showed no effect on enhancing Mbd3 occupancy at these neural differentiation-associated genes. These results suggested that activation of canonical Wnt signaling enhances Mdb3 interaction with its target neurogenesis-associated genes.

Next, we undertook ChIP–qPCR with antibodies against HDAC1 and MTA1, key components of the NuRD complex (Fig. 6b, c). Consistent with what was observed in Mbd3 enrichments, Wnt3a ligands efficiently recovered HDAC1 and MTA1 occupancy levels on the five neuronal gene loci after NPC differentiation compared with the control (by 3.3-, 2.5-, 4.7-, 5.5- and 3.1-fold, respectively, in *Dlx1*, *Ascl1*, *Tlx3*, *NeuroD1* and *Lbx1* genes). Meanwhile, DKK1 treatment significantly reduced this enhancement and, when combined with Wnt3a, resulted in occupancy rates higher than those observed in DKK1 alone but lower than those in the Wnt3a group. Similar patterns of Mbd3, HDAC1 and MTA1 enrichment at each locus under distinct canonical Wnt stimuli indicated the role of Mbd3 in facilitating the recruitment of NuRD complex subunits to specific regions of neurogenesis-associated genes. The activation of noncanonical Wnt signaling by Wnt5a, meanwhile, showed no significant promoting effect on HDAC1 and MTA1 binding, indicating that it does not contribute to the inhibition of NPC differentiation via Mbd3–NuRD complex formation. From these results, we propose the mechanism by which the canonical Wnt–Mbd3 axis regulates NPC neurogenesis (Fig. 6d). Activation of the canonical Wnt cascade via the Wnt3a ligand promotes Mbd3 stabilization by attenuating polyubiquitination, thereby preventing proteasomal degradation. The stability of Mbd3 facilitates its nuclear localization, where it binds to the promoter or gene body region of specific neurogenesis-associated genes. This facilitates the formation of the NuRD complex, which ultimately represses the transcription of target neuronal genes and thereby inhibits the neural differentiation potential of NPCs. Together, our findings define canonical Wnt signaling activated by Wnt3a as a stimulus for Mbd3 protein stabilization, enabling its functions as a co-repressor within the NuRD complex to deregulate the transcription of neurogenesis-associated genes, thereby maintaining the stemness of NPCs.

### GSK3β inhibition promotes enrichment of the Mbd3–NuRD complex on neurogenesis-associated genes

**Fig. 7:**
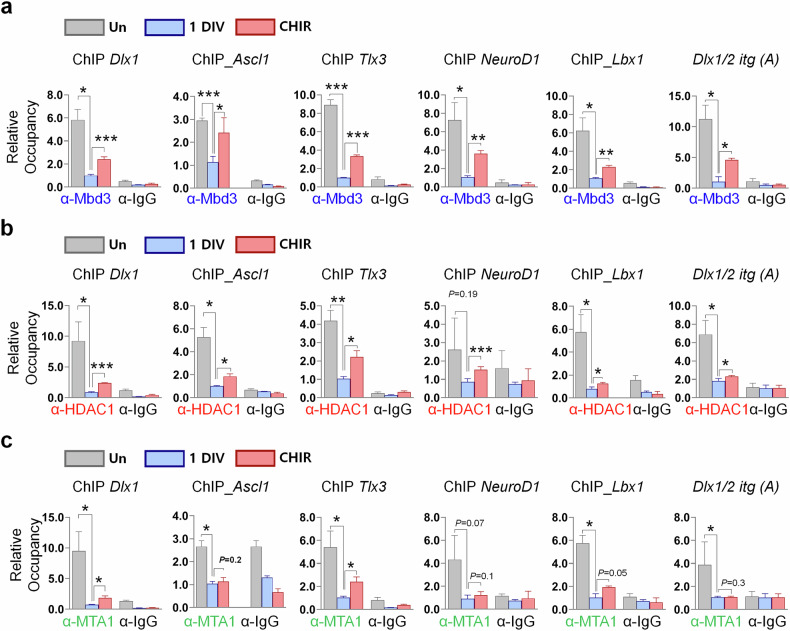
Mbd3 stabilization by GSK3β inhibitor (CHIR) treatment promotes recruitment of Mbd3–NuRD transcription-repressive complex components on neurogenesis-asociated gene loci. **a**–**c**, ChIP–qPCR analysis of Mbd3 (**a**), HDAC1 (**b**) and MTA1 (**c**) occupancy at Mbd3-binding loci in undifferentiated and differentiated cells with or without CHIR treatment (*N* = 3). Data are presented as mean ± s.d.; one-way ANOVA was performed to calculate the significance (*P < 0.05, **P < 0.01, ***P < 0.001).

To gain a comprehensive understanding of the regulatory effect of canonical Wnt signaling on neurogenesis through the Mbd3–NuRD complex, we induced NPC differentiation while blocking the negative regulator of this pathway with CHIR. Conducting ChIP–qPCR analysis of the six neuronal gene loci *Dlx1*, *Ascl1*, *Tlx3*, *NeuroD1*, *Lbx1* and *Dlx 1/2 itg(A)*, which induce different neuronal fates, using anti-Mbd3 antibody, we observed significantly increased levels of Mbd3 accumulation at promoter or gene body regions of these genes in CHIR-treated cells compared with the control (Fig. 7a). This suggests that GSK3β inhibition enhanced specific binding of Mbd3 to its target genes in differentiated cells. We next investigated the enrichment of HDAC1 and MTA1 on these loci and found that Wnt signaling activation through CHIR promoted the occupancy of both HDAC1 and MTA1 at most target genes (Fig. 7b, c). This suggests the functional interaction of the Mbd3–NuRD complex with specific canonical Wnt signaling-responsive genes, which subsequently restrict their transcription, thereby obstructing NPC neurogenesis. Therefore, our data suggest that the canonical Wnt–Mbd3 axis specifically deregulates neuronal cell-fate determination by enhancing NuRD activity, which suppresses the transcription of neurogenesis-associated genes and thereby preserves NPC stemness. For this reason, the lack of NuRD complex, facilitated by the inactivation of a canonical Wnt signaling pathway, is crucial for initiating and promoting neuronal differentiation of NPCs.

### Mbd3 engages with HDAC1 to orchestrate the recruitment of the Mbd3–NuRD complex on neurogenesis-associated genes

**Fig. 8:**
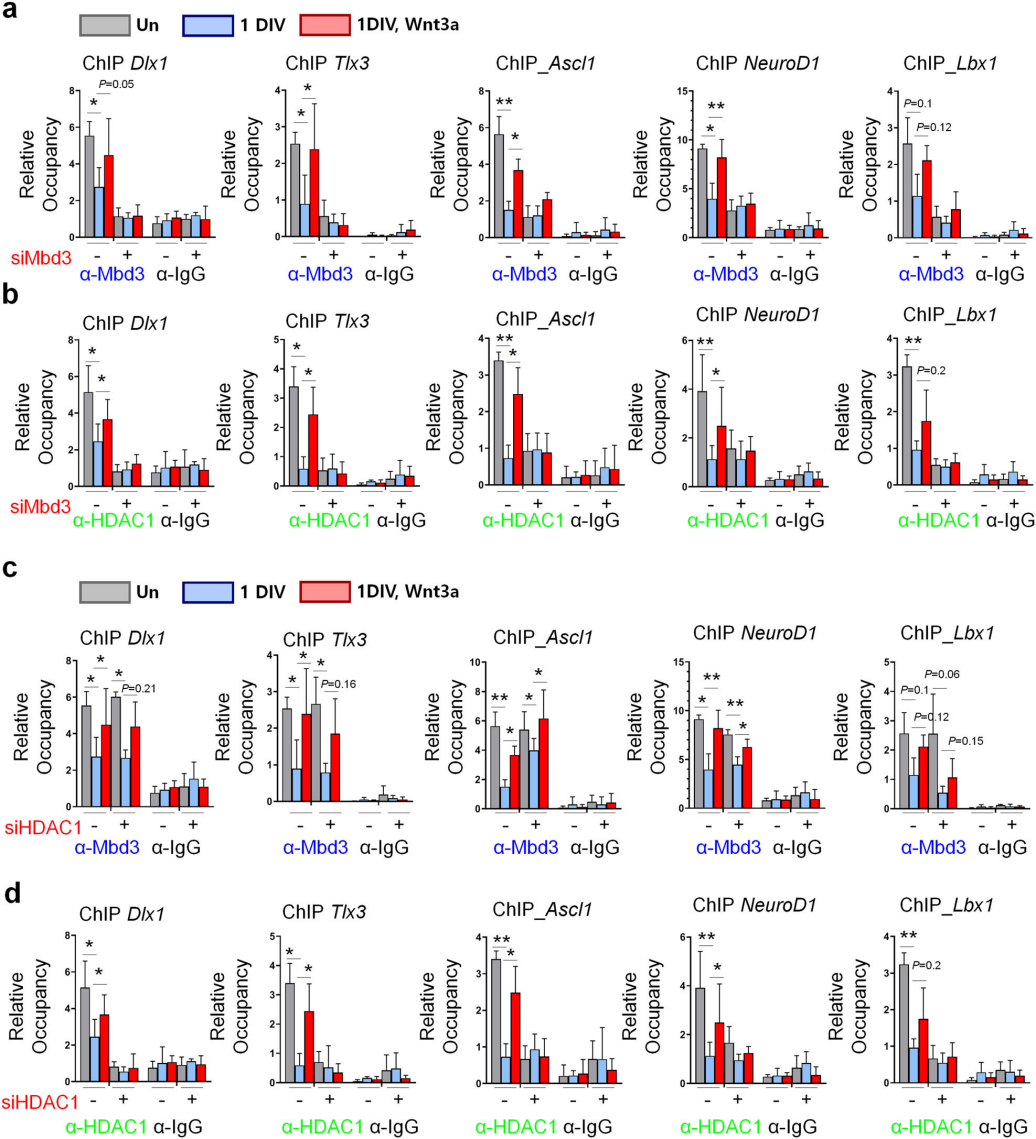
Enrichment of the Mbd3–NuRD complex on neurogenesis-associated genes is facilitated by Mbd3–HDAC1 interaction. **a**, **b** Reciprocal ChIP–qPCR targeting Mbd3 (**a**) and HDAC1 (**b**) in Mbd3-knockdown cells, with or without Wnt3a supplementation (*N* = 3). **c**, **d** Reciprocal ChIP–qPCR targeting Mbd3 (**c**) and HDAC1 (**d**) in HDAC1-knockdown cells, with or without Wnt3a supplementation (*N* = 3). Data are presented as mean ± s.d.; one-way ANOVA was performed to calculate the significance (*P < 0.05, **P < 0.01, ***P < 0.001).

The activation of the canonical Wnt–β-catenin signaling pathway enhanced the enrichment of the Mbd3–NuRD complex at target neurogenesis-associated gene loci. This prompted us to further investigate the correlation between Mbd3 and HDAC1, a key component of the NuRD complex, by assessing the effects of silencing either gene in the presence and absence of Wnt3a. Specifically, ChIP–qPCR showed that Mbd3 occupancies at the six target gene loci were substantially reduced in the siMbd3-treated group and were rescued by Wnt3a supplementation (Fig. 8a). Concurrently, the levels of HDAC1 occupying the six target neuronal gene loci were markedly reduced in siMbd3-transfected cells. However, Wnt3a treatment restored HDAC1 enrichment levels (Fig. 8b), suggesting that Mbd3 influences HDAC1 occupancy and highlighting the role of Wnt signaling activation in promoting the recruitment of the Mbd3–NuRD complex.

We next investigated Mbd3 and HDAC1 occupancy rates on neurogenesis-associated gene loci in HDAC1 silencing conditions. ChIP–qPCR analysis demonstrated that HDAC1 knockdown does not interfere with Mbd3 occupancy on target genes, compared with the scramble vector groups (Fig. 8c). HDAC1 accumulation at target loci in 1DIV differentiated NPCs reduced after HDAC1 siRNA transfection, and occupancy rates were rescued in certain genes following Wnt3a treatment (Fig. 8d), confirming the role of the Wnt signaling pathway activation in Mbd3–HDAC1 interaction. To further validate the role of Mbd3 in NuRD subunits assembly, we performed IP and detected interactions between Mbd3 and representative NuRD components, including HDAC2, MTA1, RbAP46 and CHD3 (Supplementary Fig. 3). These interactions were notably enhanced under Wnt3a treatment, confirming Mbd3’s role as a key organizer in NuRD complex formation and suggesting that Wnt signaling promotes Mbd3’s binding affinity to NuRD components. Overall, these data demonstrated that modulation of Mbd3 levels altered HDAC1 occupancy on target neuronal gene loci promoters, but HDAC1 silencing could not interfere with Mbd3 enrichment at the same positions, verifying our hypothesis on the scaffolding function of Mbd3 proteins in NuRD complex formation. Mbd3–HDAC1 interactions were validated in differentiation of NPCs with and without Wnt3a, supporting our hypothesis on the role of canonical Wnt signaling in Mbd3 stabilization, which, in turn, preserves NPC stemness through the deregulatory effect of the recruited NuRD complex.

## Discussion

Here, we elucidated a correlation between Mbd3 protein levels and the regulation of canonical Wnt signaling, focusing on Wnt3a as an activator and using β-catenin, the downstream effector of the pathway, as a reference. We found parallel alterations in the nuclear localization level of β-catenin and Mbd3 proteins in 2DIV cells corresponding to the conditional activation or disruption of Wnt signaling, suggesting that these two proteins share the same regulatory mechanism by canonical Wnt signaling. The dynamic alterations in Mbd3 expression and its polyubiquitination corresponding to each activator or inhibitor of the canonical Wnt pathway indicate that the β-catenin–Wnt pathway governs Mbd3 stabilization. Further characterizing this relationship through gain- and loss-of-function approaches targeting GSK3β—a critical regulator of the pathway that primes β-catenin for ubiquitin-mediated degradation—we observed that modulating GSK3β levels significantly influenced Mbd3–ubiquitin interactions in 2DIV cells. These interactions, in turn, conditionally directed NPCs toward proliferation or differentiation. In addition, co-IP assay detected GSK3β–Mbd3 interaction under physiological conditions, suggesting their association in Wnt signaling. These results highlight the role of GSK3β in mediating Mbd3 stability within the canonical Wnt pathway.

In the context of the NPC regulatory network, we found that the occupancies of Mbd3, HDAC1 and MTA1 on neurogenesis-associated genes altered in response to conditional activation or inhibition of canonical Wnt signaling. Notably, reciprocal ChIP–qPCR targeting Mbd3 and HDAC1 under Mbd3 or HDAC1 loss-of-function conditions revealed that Mbd3 influences HDAC1 occupancy on target genes and that this interaction could be modulated by Wnt–β-catenin signaling. Knockdown of HDAC1 did not impede Mbd3 binding to its neuronal gene loci partners, highlighting the critical role of Mbd3 in regulating NuRD complex formation. The increased interactions between Mbd3 and key NuRD complex subunits under Wnt3a treatment highlight the crucial role of Mbd3 in regulating NuRD complex assembly through the Wnt–Mbd3 cascade. These findings propose a regulatory mechanism for NPC differentiation mediated by canonical Wnt signaling and the Mbd3–NuRD complex. Activation of the canonical Wnt pathway enhances Mbd3 stability, promoting its nuclear translocation, where it facilitates the recruitment of NuRD suppressor components to the promoters or gene bodies of specific neurogenesis-associated genes. This assembly suppresses the transcription of these genes, thereby maintaining the NPC pool. Conversely, when canonical Wnt signaling is inactive, Mbd3 undergoes ubiquitin-mediated proteasomal degradation, which prevents the assembly of functional NuRD complex, permitting the transcription of genes essential for neuronal fate determination, ultimately enhancing NPC differentiation.

The acquisition of an efficient neuron network necessitates the maintenance of NPC homeostasis in conjunction with functional neural circuits^37^. Given that NPCs, characterized by self-renewal and differentiation potential, are essential for brain plasticity throughout life, a proper progression of neurogenesis and concomitant preservation of the stem cell pool are two parallel indispensable processes. Indeed, excessive differentiation of NPCs resulting from disrupted maintenance of the quiescent pool leads to depletion of precursor cells. Also, when lineage commitment fails due to impaired differentiation, new neurons cannot be generated even in the presence of a stem cell reservoir. Emerging evidence has shown that impaired NPC homeostasis disrupts tissue neurogenesis and regenerative demands and leads to neurological diseases^31,32^, emphasizing the significance of this balance as well as the mechanisms governing this control. Among the pathways that have been identified to contribute to NPC homeostasis, canonical Wnt signaling is considered essential in virtue of its cell context- and stage-specific actions^38–41^ depending on the different interactions of Wnt, its agonists or its antagonists with various receptors and co-receptors available in the cells^11,42^. Specifically, the canonical Wnt cascade stimulates self-renewal capacity and maintains neural progenitors during early neurogenesis^38^ while promoting differentiation of intermediate progenitors during mid and late neurogenesis^22^, which both are implemented by the downstream molecules β-catenin. Along with canonical Wnt, Mbd3, which is more highly expressed in the brain than in other tissues, has been demonstrated to function as an epigenetic regulator in NPCs by triggering the aggregation of the NuRD complex at gene loci associated with neurogenesis, thereby generating signals repressive to neural differentiation through inhibiting the transcription of target genes^35,36^. Deletion of the Mbd3 gene in NPCs leads to the formation of deep and upper-layer neurons^26^, implicating the requirement of Mbd3 to maintain the progenitor reservoir during neural development. In accordance with these findings, here we highlighted the profound role of Mbd3 in providing a scaffold for site-specific NuRD complex formation, which acts to keep genes required for neural differentiation in check, so that progenitor cells can respond to developmental cues to maintain appropriate transcription. Furthermore, the acquisition of this effect requires Mbd3 stability, for which several regulatory mechanisms have been identified^21,30^. In addition to these explorations, here the similar expression pattern of Mbd3 and β-catenin under different synergistic and antagonistic stimuli of canonical Wnt signaling suggest that the Wnt–β-catenin pathway regulates the maintenance of Mbd3 levels. Also, upon the inhibition of canonical Wnt cascades, both proteins undergo ubiquitin-mediated proteasomal degradation, which consolidates our hypothesis that Mbd3 is a downstream molecule of the canonical Wnt signalomes. Moreover, the increased proliferative and stemness markers, along with the enhanced generation of early and mature neurons in differentiated NPCs, corresponding respectively to the activation and inhibition of the upstream canonical Wnt pathway, suggest an overlapping role of Mbd3 and β-catenin as transcription regulators, in balancing NPC maintenance and differentiation during the early stage of neurogenesis. This similar effect, however, is not functionally redundant. Indeed, β-catenin enhances the expression of self-renewal and stemness-related genes to promote multipotent potential of the NPCs, whereas Mbd3 suppresses the transcription of neurogenesis-associated genes, hence preserving the progenitor’s reservoir. The distinct gene targets of each molecule, therefore, augment the overall effect of the canonical Wnt cascade on maintaining NPC homeostasis, which is crucially important for normal development and behaviors of the brain. For this reason, our finding that Mbd3 is a downstream compartment of the canonical Wnt signalosome emphasizes the critical coordination of extrinsic signal (Wnt and their antagonists/agonists) and intrinsic transcriptional factors (Mbd3, β-catenin) to ensure adequate balance between self-renewal and neurogenesis of NPCs. Despite the divergence in downstream targets, Wnt–β-catenin and Wnt–Mbd3 branches both direct neuronal cell-fate determination in NPCs, giving the canonical Wnt pathway an eminent status as a target not only for driving neural stem cell reprogramming and directing neuronal lineage commitment, but also for advancing the control of cellular senescence to efficiently eradicate glioblastoma malignancy.

Taken together, we identified a mechanism for regulating NPC neurogenesis through the canonical Wnt–Mbd3 axis, where Wnt activation stabilizes Mbd3, enabling it to function downstream as a transcriptional co-repressor. This process ultimately inhibits neuronal programs and promotes the stemness of NPCs. Our results establish the canonical Wnt–Mbd3 axis as a critical determinant of NPC homeostasis, working alongside the conventional Wnt–β-catenin pathway to maintain a proper balance between NPC proliferation and neuronal lineage commitment in the brain. This Wnt–Mbd3 branch provides new insights into the neurogenesis regulatory circuit governed by canonical Wnt signaling, paving the way for potential improvements in therapeutic strategies for treating neurodegenerative diseases and glioblastoma through regenerative approaches.

## Supplementary information

Supplementary Information

## Data Availability

All data supporting the findings of this study are available within the Article and its Supplementary Information.
