# Supplementary material for: Canonical Wnt signaling regulates Mbd3 protein stability during neurogenesis
Source: Exp Mol Med. 2025 Aug 1;57(8):1727–42. doi: 10.1038/s12276-025-01510-4 (PMC12411611; doi:10.1038/s12276-025-01510-4)
Supplement: Supplementary file 1 — Supplementary Information [file 12276_2025_1510_MOESM1_ESM.pdf]

Canonical Wnt signaling regulates Mbd3 protein stability during neurogenesis

Nhu Thi Quynh Mai, Soyoung Jeon, Byoung-San Moon✉

SUPPLEMENTARY INFORMATION

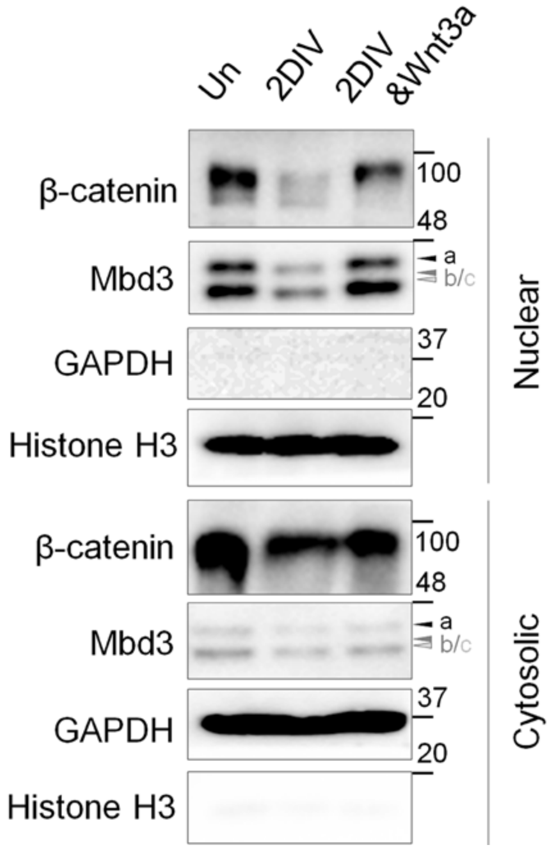

Supplementary Fig. 1: Nuclear and cytoplasmic expression of β-catenin and Mbd3 in different states of NPCs. a,b, and **c** denote Mbd3 isoforms.

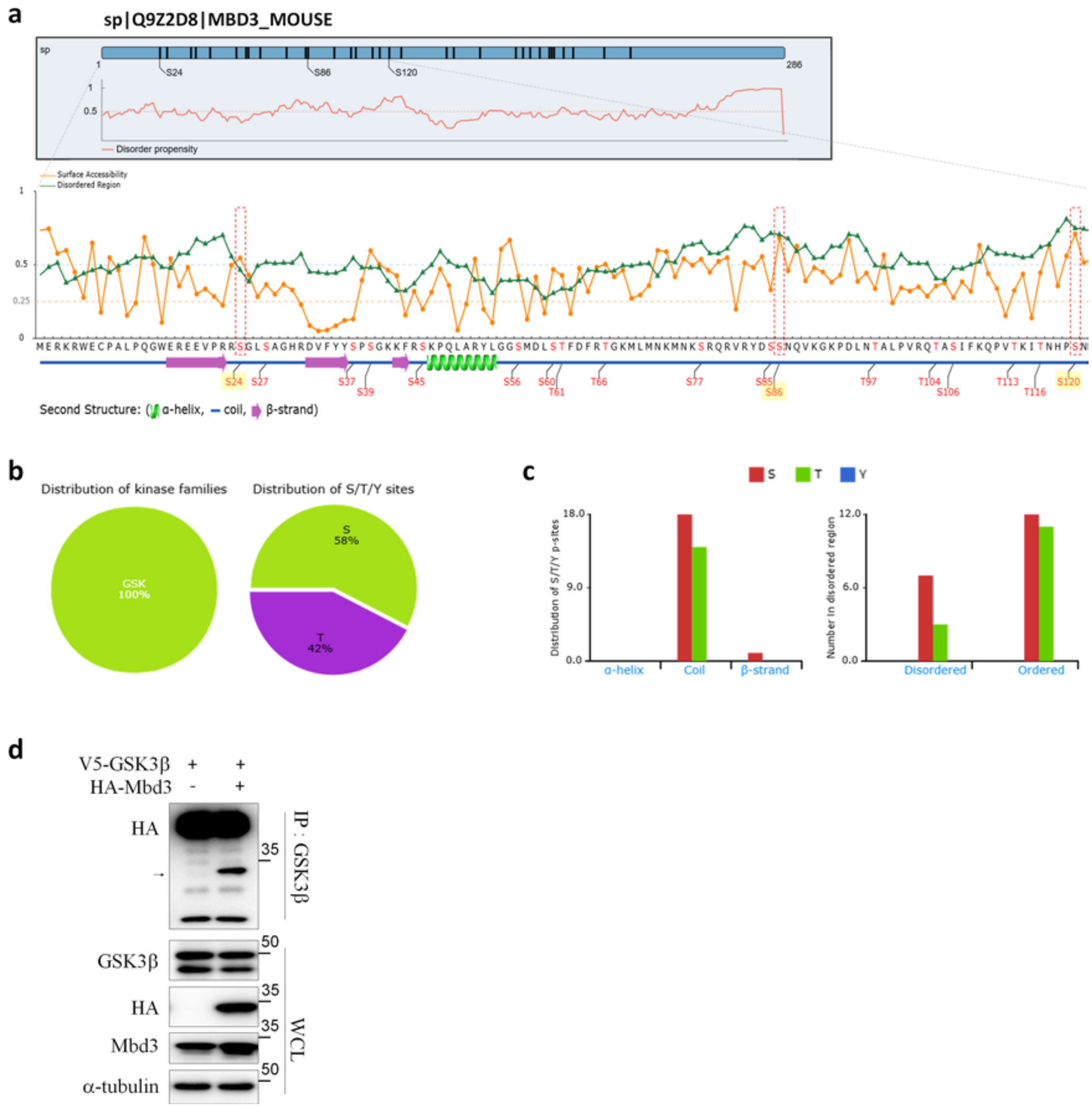

Supplementary Fig. 2: GSK3β-Mbd3 interactions in NPCs.

a (Upper) Positional distribution of the predicted Serine (S) / Threonine (T) phosphorylation sites on mouse Mbd3 by GSK3β. **(Lower)** Visualization of predicted surface accessibility, disordered region, secondary structure of mouse. **b** The predicted distribution of S/T/Y(Tyrosine) sites in kinase families (left), S/T/Y sites (right). **c** Predicted S/T/Y sites in secondary structure (middle right) and disordered region (right) of Mbd3. **d** Co-immunoprecipitation assay of 2DIV NPCs demonstrating the interactions between Mbd3 and GSK3β.

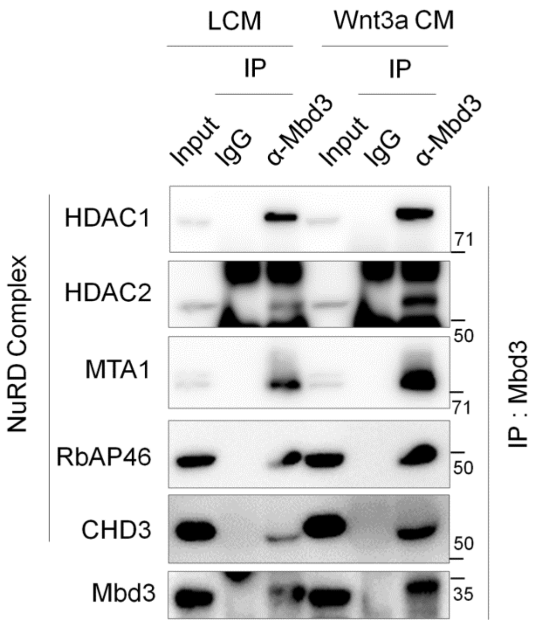

Supplementary Fig. 3: Co-immunoprecipitation assay of 2DIV NPCs in the control and Wnt3a conditions demonstrating the interactions between Mbd3 and major components of NuRD complex.

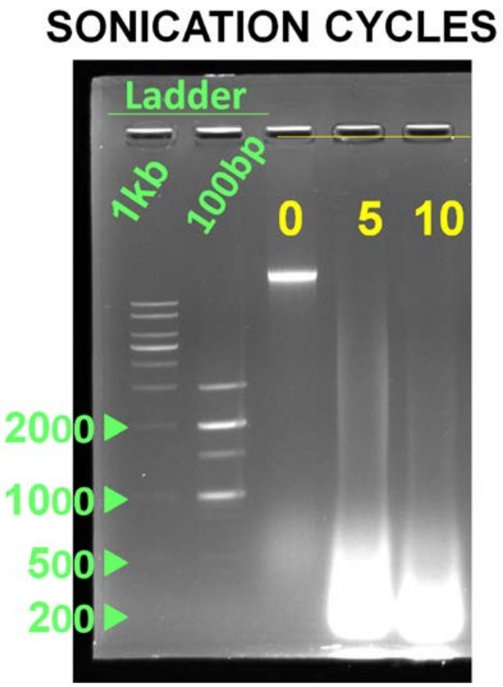

Supplementary Fig. 4: Resultant DNA fragments obtained by different sonication cycles.

Supplementary Table 1: Primer sequences used for ChIP-qPCR

Genes	Primer Sequences
Dlx1	5'-CTCTGCCAAAATGTGCTTGA-3’
	5'-CTTCCCCTATCTGGAGTGCA-3’
Dlx2	5'-TTTAAATCAATTGCCCGT-3'
	5'-ACAAGCACTGCAGAAAGTGG-3'
Lbx1	5'-ACTTCTCTCGCTGGGGAAA-3'
	5'-TCCTGCGGTCTAGAAGCTGT-3'
Tlx3	5'-CTATGCCCTCGGTGCCCACG-3'
	5'-CGGCCGCCGCTGTGAAGC-3'
NeuroD1	5'-GAAGTTGCCATTGATGCTGA-3
	5'-CGGTGCATCCCTACTCCTAC-3'
Ascl1	5'-GGAAAGGGAAAAGGAAAGCA-3
	5'-GAAAGGGGGTTCAACCAAAT-3'

Supplementary Table 2: Primer sequences used for RT-qPCR

Genes	Primer Sequences
β-catenin	5'-ACGTGCAATTCCTGAGCTGA-3’
	5'-GACAGCACCTTCAGCACTCT-3’
Mbd3	5'-GCCACAGGGATGTCTTTTACT-3'
	5'-TCCTGCTTCCTGATGTCGTC-3'
GAPDH	5'-GGCAAATTCAACGGCACAGT-3'
	5'-GACGGACACATTGGGGGTAG-3'
